# Application of insecticides by soil drenching before seedling transplanting combined with anti-insect nets to control tobacco whitefly in tomato greenhouses

**DOI:** 10.1038/s41598-022-20294-5

**Published:** 2022-09-24

**Authors:** Liangang Mao, Lan Zhang, Shaoli Wang, Yanning Zhang, Lizhen Zhu, Hongyun Jiang, Xingang Liu

**Affiliations:** 1 Institute of Plant Protection, State Key Laboratory for Biology of Plant Disease and Insect Pests, Chinese Academy of Agricultural Sciences, Beijing, 100193 China; 2 Institute of Vegetables and Flowers, Chinese Academy of Agricultural Sciences, Beijing, 100081 China

**Keywords:** Zoology, Entomology

## Abstract

Application of chemical pesticides is currently the main effective method to control tobacco whitefly (*Bemisa tabaci*) in tomato in China. The *B. tabaci* control efficacy of three systemic insecticides (thiamethoxam, sulfoxaflor and cyantraniliprole) by pre-transplant soil drenching with anti-insect nets throughout the tomato growth period was evaluated in two tomato greenhouses in the suburbs of Beijing, China, in 2018 and 2019. In two greenhouse trials, thiamethoxam 25% water dispersible granules (WDG) at a field rate of 21 g a.i./hm^2^, sulfoxaflor 22% aqueous suspension (AS) at 18 g a.i./hm^2^ or cyantraniliprole 10% oil-based suspension concentrate (OD) at 18 g a.i./hm^2^ applied via soil drenching before seedling transplanting in combination with white anti-insect nets (50 mesh) all effectively controlled the damage to *B. tabaci* and resulted in a low density of adults and eggs during the entire growing season, which was significantly lower than application of thiamethoxam, sulfoxaflor or cyantraniliprole via soil drenching before seedling transplanting without anti-insect net treatments or anti-insect nets alone (*P* < 0.05). All of the above treatments provided significantly better results than the untreated control (*P* < 0.05). All chemically treated tomato fruits had acceptable insecticide residuals that were lower than the corresponding maximum residue limits. The results suggest that application of thiamethoxam 25% WDG at a field rate of 21 g a.i./hm^2^, sulfoxaflor 22% AS at 18 g a.i./hm^2^ or cyantraniliprole 10% OD at 18 g a.i./hm^2^ by pre-transplant soil drenching combined with anti-insect nets could be recommended to control *B. tabaci* throughout the tomato growth period as part of integrated pest management programs in China.

In 2020, the total tomato (*Solanum lycopersicum* L.) area harvested in China, the largest tomato-producing country in the world, was 1,111,480 hectares (ha), and the production quantity was 64,865,807 tonnes (t)^1^. The tobacco whitefly, *Bemisa tabaci* (Hemiptera: Aleyrodidae), is a complex of biotypes^2–4^ or a complex of distinct cryptic species^5–8^ that causes worldwide damage to tomatoes^9,10^. As one of the sap-feeding insect species, *B. tabaci* causes serious direct damage, such as plant wilt, by depriving the plants of sap, indirectly damaging photosynthesis through honeydew contamination, or causing serious yield loss through the transmission of several plant pathogenic viruses^11,12^. Therefore, there is a practical need to find an effective and economically acceptable management method for *B. tabaci* control in tomato production.

There is much information in the literature on the effects of various methods, including chemical control, biological control, physical control, and resistant varieties that have been evaluated for *B. tabaci* control worldwide^13–17^. Application of chemical pesticides has been the main effective direct method used to control *B. tabaci*^16^. Currently, eight single-chemical insecticides, thiamethoxam, cyantraniliprole, dinotefuran, flupyradifurone, spirotetramat, clothianidin, buprofezin, and afidopyropen, are registered for *B. tabaci* control in tomato production in China^18^. The majority of the recommended application methods of the above single-chemical insecticides are conventional foliar spraying, and only cyantraniliprole is also registered with another method, seedbed spraying. The spraying of neonicotinoid pesticides is becoming increasingly severely restricted in tomato, especially in the flowering period, because of the negative effects on bees^19^.

Since systemic insecticides, such as neonicotinoids, can be translocated throughout plants via root systems, they can also be applied through soil drenching, which saves labor and decreases the negative effects on bees compared with conventional foliar spraying^20^. To achieve early prevention, soil drenching with systemic insecticides before seedling transplanting is considered to be a promising method for *B. tabaci* control in tomato production^21^. In recent years, chemical pesticide reduction programs have been promoted to improve the environment in China, the United Kingdom and other countries^22,23^. The combined use of chemical pesticide control and other control methods such as anti-insect nets is a promising method to reduce the use of chemical pesticides.

The objective of this study was to identify the efficacy and feasibility of soil drenching with three systemic insecticides (thiamethoxam, sulfoxaflor and cyantraniliprole) before seedling transplanting in combination with anti-insect nets for *B. tabaci* control throughout the tomato growth period. The study also aimed to evaluate the food safety of the first-harvest fruit.

## Results

### Control efficacy on *B. tabaci* adults

#### Trial I

*B. tabaci* adults were first observed on the plants in the untreated control plots at 9 weeks after transplanting (WAT); however, there was no significant difference in the populations between the untreated control plots and the other treated plots (*F*_7, 24_ = 8.999, *P* = 0.378) (Table 1). Then, the populations of adults in untreated control plots increased quickly, maintaining a high density from 12 WAT until the end of the trials. No *B. tabaci* adults were observed in the plots treated alone with thiamethoxam, sulfoxaflor, cyantraniliprole or anti-insect nets until 12 WAT. Thiamethoxam & net, sulfoxaflor & net and cyantraniliprole & net treatments all effectively delayed the occurrence of *B. tabaci* adults until 15 WAT and reduced the number of adults by at least 89% during the whole growing period (Table 1).

**Table 1:** Effects of different treatments on *Bemisa tabaci* adult populations during the whole growing season (greenhouse trial I, 2018).

Treatment	1WBT^a^	0WAT^b^	1WAT	4WAT	9WAT	12WAT		15WAT		18WAT	
	No./plant	No./plant	No./plant	No./plant	No./plant	No./plant	% Reduction	No./plant	% Reduction	No./plant	% Reduction
Thiamethoxam & net	0.0 ± 0.0a^c^	0.0 ± 0.0a	0.0 ± 0.0a	0.0 ± 0.0a	0.0 ± 0.0a	0.0 ± 0.0d	100	1.3 ± 0.3e	99.0	4.1 ± 1.8c	92.9
Sulfoxaflor & net	0.0 ± 0.0a	0.0 ± 0.0a	0.0 ± 0.0a	0.0 ± 0.0a	0.0 ± 0.0a	0.0 ± 0.0d	100	0.8 ± 0.3e	99.4	3.0 ± 0.9c	94.8
Cyantraniliprole & net	0.0 ± 0.0a	0.0 ± 0.0a	0.0 ± 0.0a	0.0 ± 0.0a	0.0 ± 0.0a	0.0 ± 0.0d	100	13.1 ± 2.8d	89.9	4.2 ± 1.8c	92.8
Net alone	0.0 ± 0.0a	0.0 ± 0.0a	0.0 ± 0.0a	0.0 ± 0.0a	0.0 ± 0.0a	1.0 ± 1.3 cd	95.1	38.4 ± 8.1b	70.5	16.0 ± 7.1b	72.4
Thiamethoxam alone	0.0 ± 0.0a	0.0 ± 0.0a	0.0 ± 0.0a	0.0 ± 0.0a	0.0 ± 0.0a	0.8 ± 0.3d	96.1	17.5 ± 5.9 cd	86.6	15.5 ± 7.5b	73.3
Sulfoxaflor alone	0.0 ± 0.0a	0.0 ± 0.0a	0.0 ± 0.0a	0.0 ± 0.0a	0.0 ± 0.0a	5.8 ± 5.0bc	71.6	22.2 ± 8.4bcd	82.9	19.3 ± 7.9b	66.7
Cyantraniliprole alone	0.0 ± 0.0a	0.0 ± 0.0a	0.0 ± 0.0a	0.0 ± 0.0a	0.0 ± 0.0a	9.3 ± 9.0b	54.4	29.0 ± 19.1bc	77.7	13.5 ± 3.9b	76.7
Untreated control	0.0 ± 0.0a	0.0 ± 0.0a	0.0 ± 0.0a	0.0 ± 0.0a	0.1 ± 0.1a	20.4 ± 7.4a	–	130.2 ± 56.0a	–	58.0 ± 38.3a	–

^a^WBT, weeks before transplanting; 1 WBT is the time of root drenching.

^b^WAT, weeks after transplanting; 0 WAT is the time of seedling transplanting; 12 WAT is the time of first fruit harvest; 18 WAT is the end of the greenhouse trials.

^c^In the columns, the data are the means ± SD of four replications, with 10 plants per replicate. Means followed by the same letter are not significantly different at *P* = 0.05 level according to the LSD test.

At 15 WAT, both the sulfoxaflor & net and thiamethoxam & net treated plots showed a reduced number of adults by at least 99% and the lowest density of adults on plants (0.8 and 1.3 adults per plant, respectively), which was significantly lower than that of the cyantraniliprole & net treatment plots (13.1 adults per plant) (*F*_2, 9_ = 139.627, *P* < 0.001). The adult density in the net-only treatment plots was similar to that in the sulfoxaflor-only and cyantraniliprole-only treatment plots (*F*_2, 9_ = 1.588, *P* = 0.257), but lower than that of untreated control plots (*F*_1, 6_ = 15.994, *P* = 0.007). At the end of the trial, the thiamethoxam & net, sulfoxaflor & net and cyantraniliprole & net treatments showed a reduced number of adults by at least 92%, and all had the lowest adult density, which was significantly lower than that of thiamethoxam-only, sulfoxaflor-only, cyantraniliprole-only and net-only treatments (*F*_6, 21_ = 10.376, *P* < 0.003); all of the above treatments maintained a significantly lower adult density than the untreated control (*F*_7, 24_ = 10.903, *P* < 0.001) (Table 1).

#### Trial II

No *B. tabaci* adults were observed on the plants in all plots from 1 week before transplanting (WBT) to 7 WAT. At 11 WAT, there were still no tobacco whitefly adults on plants in the thiamethoxam & net, sulfoxaflor & net and cyantraniliprole & net treatment plots, which was similar to that in the net-only treatment plot at *P* = 0.05 level, but significantly lower than that in the thiamethoxam-only, sulfoxaflor-only, and cyantraniliprole-only treatment plots (*F*_5, 18_ = 24.738, *P* < 0.01); all the treatments showed a lower adult density than that of the untreated control plots (*F*_7, 24_ = 33.686, *P* < 0.003) (Table 2). The thiamethoxam & net, sulfoxaflor & net and cyantraniliprole & net treatments all effectively delayed the occurrence of *B. tabaci* adults until 14 WAT and reduced the number of adults by at least 98% during the whole growing period (Table 2).

**Table 2:** Effects of different treatments on *Bemisa tabaci* adult populations during the whole growing season (greenhouse trial II, 2019).

Treatment	1WBT^a^	0WAT^b^	1WAT	4WAT	7WAT	11WAT		14WAT		18WAT	
	No./plant	No./plant	No./plant	No./plant	No./plant	No./plant	% reduction	No./plant	% reduction	No./plant	% reduction
Thiamethoxam & net	0.0 ± 0.0a^c^	0.0 ± 0.0a	0.0 ± 0.0a	0.0 ± 0.0a	0.0 ± 0.0a	0.0 ± 0.0d	100	0.5 ± 0.4d	99.6	7.7 ± 5.4d	98.2
Sulfoxaflor & net	0.0 ± 0.0a	0.0 ± 0.0a	0.0 ± 0.0a	0.0 ± 0.0a	0.0 ± 0.0a	0.0 ± 0.0d	100	1.6 ± 1.5d	98.8	8.1 ± 3.4d	98.1
Cyantraniliprole & net	0.0 ± 0.0a	0.0 ± 0.0a	0.0 ± 0.0a	0.0 ± 0.0a	0.0 ± 0.0a	0.0 ± 0.0d	100	0.9 ± 0.7d	99.3	3.7 ± 3.6d	99.1
Net alone	0.0 ± 0.0a	0.0 ± 0.0a	0.0 ± 0.0a	0.0 ± 0.0a	0.0 ± 0.0a	0.3 ± 0.2 cd	99.4	4.3 ± 2.2d	96.9	54.1 ± 18.4c	87.1
Thiamethoxam alone	0.0 ± 0.0a	0.0 ± 0.0a	0.0 ± 0.0a	0.0 ± 0.0a	0.0 ± 0.0a	3.3 ± 1.7c	93.9	47.9 ± 21.8c	65.2	71.3 ± 30.4bc	83.0
Sulfoxaflor alone	0.0 ± 0.0a	0.0 ± 0.0a	0.0 ± 0.0a	0.0 ± 0.0a	0.0 ± 0.0a	25.4 ± 7.6b	52.9	98.4 ± 49.1ab	28.5	137.8 ± 44.1b	67.1
Cyantraniliprole alone	0.0 ± 0.0a^c^	0.0 ± 0.0a	0.0 ± 0.0a	0.0 ± 0.0a	0.0 ± 0.0a	14.6 ± 13.3b	72.9	74.7 ± 63.9bc	45.7	126.9 ± 74.5bc	69.7
Untreated control	0.0 ± 0.0a	0.0 ± 0.0a	0.0 ± 0.0a	0.0 ± 0.0a	0.0 ± 0.0a	53.9 ± 25.9a	–	137.6 ± 61.7a	–	419.2 ± 212.9a	–

^a^WBT, weeks before transplanting; 1 WBT is the time of root drenching.

^b^WAT, weeks after transplanting; 0 WAT is the time of seedling transplanting; 11 WAT is the time of first fruit harvest; 18 WAT is the end of the greenhouse trials.

^c^In the columns, the data are the means ± SD of four replications, with 10 plants per replicate. Means followed by the same letter are not significantly different at *P* = 0.05 level according to the LSD test.

At 14 WAT, the thiamethoxam & net, sulfoxaflor & net, cyantraniliprole & net, and net-only treatment plots showed a reduced number of adults by at least 96%, and all had the lowest adult density, which was significantly lower than that in the thiamethoxam-only, sulfoxaflor-only, and cyantraniliprole-only treatment plots (*F*_6, 21_ = 20.040, *P* < 0.001); all of the above treatments except for the sulfoxaflor-only treatment showed a lower adult density than that of the untreated control plots (*F*_6, 21_ = 23.976, *P* < 0.013) (Table 2). At the end of the trial, the thiamethoxam & net, sulfoxaflor & net and cyantraniliprole & net treatments reduced the number of adults by at least 98%, and all had the lowest adult density, which was significantly lower than that of thiamethoxam alone, sulfoxaflor alone, cyantraniliprole alone and net alone (*F*_6, 21_ = 21.355, *P* < 0.002); all of the above treatments showed a significantly lower adult density than that of the untreated control (*F*_7, 24_ = 21.560, *P* < 0.001) (Table 2).

### Control effect on *B. tabaci* eggs

#### Trial I

No *B. tabaci* eggs were observed on the plants in all plots from 1 WBT to 9 WAT. At 12 WAT, there were still no *B. tabaci* eggs on plants in the thiamethoxam & net, sulfoxaflor & net, cyantraniliprole & net, and thiamethoxam-only treatment plots, which was significantly lower than that in the sulfoxaflor-only and cyantraniliprole-only treatment plots (*F*_5, 18_ = 21.502, *P* < 0.001); all of the above treatments except for the cyantraniliprole-only treatment showed a lower egg density than that of the untreated control plots (*F*_6, 21_ = 22.610, *P* < 0.001) (Table 3). The thiamethoxam & net, sulfoxaflor & net and cyantraniliprole & net treatments all effectively delayed the occurrence of *B. tabaci* eggs and reduced the number of eggs by at least 98% during the whole growing period (Table 3).

**Table 3:** Effects of different treatments on *Bemisa tabaci* egg populations during the whole growing season (greenhouse trial I, 2018).

Treatment	1WBT^a^	0WAT^b^	1WAT	4WAT	9WAT	12WAT		15WAT		18WAT	
	No./plant	No./plant	No./plant	No./plant	No./plant	No./plant	% reduction	No./plant	% reduction	No./plant	% reduction
Thiamethoxam & net	0.0 ± 0.0a^c^	0.0 ± 0.0a	0.0 ± 0.0a	0.0 ± 0.0a	0.0 ± 0.0a	0.0 ± 0.0c	100	0.4 ± 0.4 fg	99.8	0.3 ± 0.3c	99.4
Sulfoxaflor & net	0.0 ± 0.0a	0.0 ± 0.0a	0.0 ± 0.0a	0.0 ± 0.0a	0.0 ± 0.0a	0.0 ± 0.0c	100	0.0 ± 0.0 g	100	0.6 ± 0.5c	98.7
Cyantraniliprole & net	0.0 ± 0.0a	0.0 ± 0.0a	0.0 ± 0.0a	0.0 ± 0.0a	0.0 ± 0.0a	0.0 ± 0.0c	100	2.7 ± 1.0ef	98.5	0.9 ± 0.5c	98.1
Net alone	0.0 ± 0.0a	0.0 ± 0.0a	0.0 ± 0.0a	0.0 ± 0.0a	0.0 ± 0.0a	2.1 ± 1.8b	92.6	22.6 ± 22.5 cd	87.3	24.5 ± 12.8b	48.7
Thiamethoxam alone	0.0 ± 0.0a	0.0 ± 0.0a	0.0 ± 0.0a	0.0 ± 0.0a	0.0 ± 0.0a	0.0 ± 0.0c	100	9.8 ± 8.9de	94.5	34.8 ± 31.0ab	27.2
Sulfoxaflor alone	0.0 ± 0.0a	0.0 ± 0.0a	0.0 ± 0.0a	0.0 ± 0.0a	0.0 ± 0.0a	5.4 ± 4.8b	80.9	49.6 ± 45.9bc	72.2	28.3 ± 12.8ab	40.8
Cyantraniliprole alone	0.0 ± 0.0a	0.0 ± 0.0a	0.0 ± 0.0a	0.0 ± 0.0a	0.0 ± 0.0a	14.9 ± 9.6a	47.3	113.2 ± 63.3ab	36.6	25.3 ± 14.8b	47.1
Untreated control	0.0 ± 0.0a	0.0 ± 0.0a	0.0 ± 0.0a	0.0 ± 0.0a	0.0 ± 0.0a	28.3 ± 20.4a	–	178.5 ± 119.5a	–	47.8 ± 18.5a	–

^a^WBT, weeks before transplanting; 1 WBT is the time of root drenching.

^b^WAT, weeks after transplanting; 0 WAT is the time of seedling transplanting; 12 WAT is the time of first fruit harvest; 18 WAT is the end of the greenhouse trials.

^c^In the columns, the data are the means ± SD of four replications, with 10 plants per replicate. Means followed by the same letter are not significantly different at *P* = 0.05 level according to the LSD test.

At 15 WAT, there were still no *B. tabaci* eggs on plants in the sulfoxaflor & net treatment plots, which was similar to that in the thiamethoxam & net treatment plots (*F*_1, 6_ = 2.877, *P* = 0.141), but significantly lower than that in the cyantraniliprole & net-treated plots (*F*_1, 6_ = 16.816, *P* = 0.006). At the end of the trial, the thiamethoxam & net, sulfoxaflor & net and cyantraniliprole & net treatments all showed the lowest egg density (0.3, 0.6 and 0.9 eggs per plant, respectively), which was significantly lower than that of the thiamethoxam-only, sulfoxaflor-only, cyantraniliprole-only and net-only treatments (*F*_6, 21_ = 14.086, *P* < 0.001); all of the above treatments except for thiamethoxam alone and sulfoxaflor alone showed a significantly lower egg density than that of the untreated control (*F*_5, 18_ = 29.607, *P* < 0.013) (Table 3).

#### Trial II

No *B. tabaci* eggs were observed on the plants in all plots from 1 WBT to 7 WAT. At 11 WAT, there were still no *B. tabaci* eggs on plants in the thiamethoxam & net, sulfoxaflor & net and cyantraniliprole & net treatment plots, which was significantly lower than that in the thiamethoxam-only, sulfoxaflor-only, cyantraniliprole-only, and net-only treatment plots (*F*_6, 21_ = 67.808, *P* < 0.001); all of the above treatments showed a lower egg density than that of the untreated control plots (*F*_7, 24_ = 45.294, *P* < 0.001) (Table 4). The thiamethoxam & net, sulfoxaflor & net and cyantraniliprole & net treatments all effectively delayed the occurrence of *B. tabaci* eggs until 14 WAT and reduced the number of eggs by at least 99% during the whole growing period (Table 4).

**Table 4:** Effects of different treatments on *Bemisa tabaci* egg populations during the whole growing season (greenhouse trial II, 2019).

Treatment	1WBT^a^	0WAT^b^	1WAT	4WAT	7WAT	11WAT		14WAT		18WAT	
	No./plant	No./plant	No./plant	No./plant	No./plant	No./plant	% reduction	No./plant	% reduction	No./plant	% reduction
Thiamethoxam & net	0.0 ± 0.0a^c^	0.0 ± 0.0a	0.0 ± 0.0a	0.0 ± 0.0a	0.0 ± 0.0a	0.0 ± 0.0d	100	0.3 ± 0.2d	99.8	0.7 ± 0.4d	99.7
Sulfoxaflor & net	0.0 ± 0.0a	0.0 ± 0.0a	0.0 ± 0.0a	0.0 ± 0.0a	0.0 ± 0.0a	0.0 ± 0.0d	100	0.2 ± 0.2d	99.8	0.6 ± 0.5d	99.7
Cyantraniliprole & net	0.0 ± 0.0a	0.0 ± 0.0a	0.0 ± 0.0a	0.0 ± 0.0a	0.0 ± 0.0a	0.0 ± 0.0d	100	0.6 ± 0.4d	99.5	1.2 ± 0.6 cd	99.4
Net alone	0.0 ± 0.0a	0.0 ± 0.0a	0.0 ± 0.0a	0.0 ± 0.0a	0.0 ± 0.0a	2.4 ± 1.5c	97.3	11.8 ± 6.2c	90.8	34.9 ± 18.1b	83.9
Thiamethoxam alone	0.0 ± 0.0a	0.0 ± 0.0a	0.0 ± 0.0a	0.0 ± 0.0a	0.0 ± 0.0a	8.3 ± 2.6c	90.6	20.2 ± 9.3c	84.2	23.1 ± 11.3b	89.4
Sulfoxaflor alone	0.0 ± 0.0a	0.0 ± 0.0a	0.0 ± 0.0a	0.0 ± 0.0a	0.0 ± 0.0a	7.9 ± 2.4c	91.0	18.6 ± 6.3c	85.4	14.3 ± 7.4bc	93.4
Cyantraniliprole alone	0.0 ± 0.0a	0.0 ± 0.0a	0.0 ± 0.0a	0.0 ± 0.0a	0.0 ± 0.0a	22.8 ± 7.8b	74.1	38.9 ± 28.3b	69.5	16.1 ± 8.8b	92.6
Untreated control	0.0 ± 0.0a	0.0 ± 0.0a	0.0 ± 0.0a	0.0 ± 0.0a	0.0 ± 0.0a	87.9 ± 39.9a	–	127.7 ± 56.5a	–	217.1 ± 131.7a	–

^a^WBT, weeks before transplanting; 1 WBT is the time of root drenching.

^b^WAT, weeks after transplanting; 0 WAT is the time of seedling transplanting; 11 WAT is the time of first fruit harvest; 18 WAT is the end of the greenhouse trials.

^c^In the columns, the data are the means ± SD of four replications, with 10 plants per replicate. Means followed by the same letter are not significantly different at *P* = 0.05 level according to the LSD test.

At 14 WAT, the thiamethoxam & net, sulfoxaflor & net, cyantraniliprole & net-treated plots showed a reduced number of eggs by at least 99%, and all had the lowest egg density (0.3, 0.2 and 0.6 eggs per plant, respectively), which was significantly lower than that in plots treated with thiamethoxam alone, sulfoxaflor alone, cyantraniliprole alone, and net alone (*F*_6, 21_ = 26.596, *P* < 0.001); all the treatments showed a lower egg density than that of the untreated control plots (*F*_7, 24_ = 33.620, *P* < 0.001) (Table 4). At the end of the trial, the thiamethoxam & net, sulfoxaflor & net, and cyantraniliprole & net treatments showed a reduced number of eggs by at least 99%, and all had the lowest egg density (0.7, 0.6 and 1.2 eggs per plant, respectively); all of the above treatments showed a significantly lower egg density than that of the untreated control (*F*_7, 24_ = 26.978, *P* < 0.001) (Table 4).

### Residual insecticides in tomato fruits at the first harvest

The maximum residue limits of thiamethoxam, sulfoxaflor, and cyantraniliprole in tomato fruits are 1, 1.5 and 0.2 mg/kg, respectively^24^. At the first fruit harvest in trial I, the maximum detected residual concentrations of thiamethoxam were 0.0013 and 0.0015 mg/kg in the fruits of the thiamethoxam & net and thiamethoxam-only treatments; all of the detected residual concentrations of sulfoxaflor were lower than 0.0001 mg/kg in fruits of sulfoxaflor & net and sulfoxaflor-only treatments; the maximum detected residual concentrations of cyantraniliprole were 0.0011 and 0.0003 mg/kg in the fruits of the cyantraniliprole & net and cyantraniliprole-only treatments (Table 5). In trial II, the maximum detected residual concentrations of thiamethoxam were 0.0041 and 0.0023 mg/kg in the fruits of the thiamethoxam & net and thiamethoxam-only treatments, respectively; all of the detected residual concentrations of sulfoxaflor were lower than 0.001 mg/kg in the fruits of the sulfoxaflor & net and sulfoxaflor-only treatments; all of the detected residual concentrations of cyantraniliprole were lower than 0.001 mg/kg in the fruits of the cyantraniliprole & net and cyantraniliprole-only treatments (Table 5). All of the above detected residual concentrations of insecticides at the first fruit harvest were lower than the corresponding maximum residue limits.

**Table 5:** Insecticide residues in tomato fruits in different insecticide treatment plots at the time of first harvest in two greenhouse trials.

Site	Insecticide treatment	Insecticide residual concentration		
		Thiamethoxam (mg/kg)	Sulfoxaflor (mg/kg)	Cyantraniliprole (mg/kg)
Greenhouse trial I, 2018	Thiamethoxam & net	≤ 0.0013		
	Sulfoxaflor & net		< 0.0001	
	Cyantraniliprole & net			≤ 0.0011
	Thiamethoxam alone	≤ 0.0015		
	Sulfoxaflor alone		< 0.0001	
	Cyantraniliprole alone			≤ 0.0003
Greenhouse trial II, 2019	Thiamethoxam & net	≤ 0.0041		
	Sulfoxaflor & net		< 0.0001	
	Cyantraniliprole & net			< 0.0001
	Thiamethoxam alone	≤ 0.0023		
	Sulfoxaflor alone		< 0.0001	
	Cyantraniliprole alone			< 0.0001

## Discussion

The total cost of discovery and development of a new pesticide product is very expensive (approximately $286 million) and time-consuming (approximately 11.3 years)^25^. It is very important to determine more improvements in application methods to extend the application life, especially for an old pesticide active ingredient such as thiamethoxam, which was launched by Novartis in 1998^26^.

The challenge for a pesticide product is to develop a new application method that is efficient and less labor intensive^27^. Seedling box application of systemic pesticides before transplanting was previously adopted in rice in Japan as a labor-saving method with less impact on the environment^28,29^. In China, all of the application methods of the registered single-chemical insecticides for *B. tabaci* control in tomato production are foliar spraying except for cyantraniliprole, which is also registered for seedbed spraying^18^. In our greenhouse trial study, seedling tray soil drenching before seedling transplanting was applied as a novel method, which will also save labor and decrease the negative effects on bees compared with conventional foliar spraying. The field rate of cyantraniliprole in our study (18 g a.i./hm^2^) was approximately 50% lower than the lowest registered rate of cyantraniliprole for seedbed spraying (37–45 g a.i./hm^2^, using rate calculation at the same tomato crop transplanting density as in our study). In our two trials, the control efficacy of cyantraniliprole soil drenching before seedling transplanting without anti-insect nets was not stable, 45.7–77.7% on *B. tabaci* adults and 36.6–92.6% on *B. tabaci* eggs, which can be attributed to the reduced application rate of cyantraniliprole.

Anti-insect nets are a good control method to prevent flying insects from entering agriculture, which is even more important than killing them by other methods^30^. In China, anti-insect net has been widely used in protected agriculture for a long time. In our two trials, the anti-insect net alone treatments provided good control efficacy (95.1% and 99.4%) for *B. tabaci* adults and eggs (92.6% and 97.3%) in the early growth period (11 WAT or 12 WAT) of the tomatoes, which was equal to or even better than the use of systemic insecticides alone, but their control efficacy decreased sharply with the emergence of *B. tabaci* in large numbers. The combined use of chemical pesticide control and anti-insect nets is confirmed to be a promising method to improve the control efficacy and reduce the use of chemical pesticides in our studies. In the combination treatments, the combined use of anti-insect nets significantly improved the control efficacy of the systemic insecticides, which were significantly better than the systemic insecticides used alone and provided more persistent control on *B. tabaci* than anti-insect net-only treatments. In addition, ensuring no *B. tabaci* adults or eggs on the seedlings is also vital to the success of control during the whole growth period of tomato, which is also affected by the regularity of occurrence of *B. tabaci*.

Providing good control efficacy is only one aspect for the scientific application of a pesticide product, and ensuring protection of the environment and food safety is also very important. Environmental pollution issues caused by pesticides have received increasing attention in recent years^31^. Spraying of neonicotinoid pesticides, such as thiamethoxam, is becoming increasingly severely restricted in tomato, especially in the flowering period, because of the negative environmental effects, especially on bees^19^. All of the tomato plants were pollinated by bumblebee (*Bombus* spp.) in the trials, however, no bee poisoning deaths were found in any of the treated plots, providing a preliminary indication of the safety of pollination bumblebees. Based on the residual results for the three systemic insecticides in the first-harvest tomato fruits, which were all below the responding maximum residue limits, the harvested tomato fruits are safe for consumers when the three insecticides are applied via the above methods.

Almost any pesticide that is frequently applied in the field will face pest resistance problems, especially for small insects such as tobacco whiteflies, aphids and planthoppers^32^. Generally, a reduced rate of pesticide use will help decrease the development speed of pesticide resistance in insects. Therefore, our combined application of a reduced rate of systemic pesticide application and net provides a better method to retard development of resistance than blind increases the use of pesticides to achieve higher control efficacy. To solve the issue of insecticide resistance in *Bemisia tabaci*, integrated pest management (IPM) and insecticide resistance management (IRM) programs such as combinations or rational rotations of insecticides with different modes of action and nonchemical methods are needed^33^.

In summary, the combined use of systemic insecticides (thiamethoxam, sulfoxaflor or cyantraniliprole) applied via soil drenching before seedling transplanting and anti-insect nets is an effective and economically acceptable management method for *B. tabaci* control throughout the tomato growth period in China. However, additional detailed research is needed to identify the optimal application protocols—including the recommended rate and suitable combinations with biological agents such as predatory and parasitic natural enemies^14,17,34^—to clarify aspects of the combined use of anti-insect nets and soil drenching with systemic insecticides (thiamethoxam, sulfoxaflor or cyantraniliprole) in seedling trays before transplanting prior to recommendation as an effective management method for *B. tabaci* control throughout the tomato growth period in China.

## Materials and methods

### Plants

Tomato (*Solanum lycopersicum* L.) was taken as the plant material for this study. The tomatoes seeds cultivar Xianke No. 8 and cultivar Jingcai No. 6 were obtained from Jingyan Yinong (Beijing) Seed Sci-Tech Co., Ltd. and Beijing Institute of Beijing Fan Tomato, respectively.

### Insecticides and anti-insect nets

Thiamethoxam 25% water dispersible granules (WDG) were obtained from Syngenta Crop Protection Co., LTD (Switzerland). Sulfoxaflor 22% aqueous suspension (AS) was obtained from Dow AgroSciences Company (America). Cyantraniliprole 10% oil-based suspension concentrate (OD) was obtained from DuPont Company (America). White anti-insect nets (50 mesh) were obtained from JC Pacific International Co., Ltd. (China).

### Greenhouse trials

#### Trials location and design

During 2018 and 2019, two demonstration experiments were conducted in tomato greenhouses on commercial farms. The farm location for greenhouse trials I and II was in Changping District, Beijing, China (40° 08′ 35.08′′ N, 116° 20′ 42.96′′). The farm has grown various vegetables, including tomatoes, cucumbers and peppers, for a long time and has been heavily infested by *B. tabaci* for many years.

In greenhouse trials I and II, all treatments in each trial were performed in randomized blocks with four replicates (Table 6). Each plot was 50 m^2^ (6.25 m wide by 8 m long). In greenhouse trial I, the tomatoes (cultivar Xianke No. 8) were sown on 16 January 2018 and transplanted on 24 March 2018; in greenhouse trial II, the tomatoes (cultivar Jingcai No. 6) were sown on 27 February 2019 and transplanted on 2 April 2019. The tomato crop transplant density in both greenhouse trials was 112 plants per plot, an equivalent of 22,400 plants/hm^2^. All tomato seeds were sown in 72-cell trays (approximately 475 cells/m^2^), and the seedlings were carefully cultivated in a seedling greenhouse before seedling transplanting.

**Table 6:** Experimental greenhouse trial treatments.

Sites	Treatment	Pesticide	Anti-insect net	Field rate (g a.i./hm^2^)	Application method
Greenhouse trial I, 2018	Thiamethoxam & net	Thiamethoxam 25% WDG	Yes	21	Soil drenching before transplanting
	Sulfoxaflor & net	Sulfoxaflor 22% AS	Yes	18	Soil drenching before transplanting
	Cyantraniliprole & net	Cyantraniliprole 10% OD	Yes	18	Soil drenching before transplanting
	Net alone	Water control	Yes	–	–
	Thiamethoxam alone	Thiamethoxam 25% WDG	No	21	Soil drenching before transplanting
	Sulfoxaflor alone	Sulfoxaflor 22% AS	No	18	Soil drenching before transplanting
	Cyantraniliprole alone	Cyantraniliprole 10% OD	No	18	Soil drenching before transplanting
	Untreated control	Water control	No	–	–
Greenhouse trial II, 2019	Thiamethoxam & net	Thiamethoxam 25% WDG	Yes	21	Soil drenching before transplanting
	Sulfoxaflor & net	Sulfoxaflor 22% AS	Yes	18	Soil drenching before transplanting
	Cyantraniliprole & net	Cyantraniliprole 10% OD	Yes	18	Soil drenching before transplanting
	Net alone	Water control	Yes	–	–
	Thiamethoxam alone	Thiamethoxam 25% WDG	No	21	Soil drenching before transplanting
	Sulfoxaflor alone	Sulfoxaflor 22% AS	No	18	Soil drenching before transplanting
	Cyantraniliprole alone	Cyantraniliprole 10% OD	No	18	Soil drenching before transplanting
	Untreated control	Water control	No	–	–

Three systemic insecticides (thiamethoxam, sulfoxaflor and cyantraniliprole) were applied via soil drenching approximately one week before seedling transplanting. Thiamethoxam at a field rate of 21 g a.i./hm^2^ (converted into a tray rate of approximately 4453 g a.i./hm^2^) was applied as follows: thiamethoxam 25% WDG was diluted approximately 1600 times with water, and each plant was soil-drenched with 6 mL of the solution. Sulfoxaflor at a field rate of 18 g a.i./hm^2^ (converted into a tray rate of approximately 3817 g a.i./hm^2^) was applied as follows: sulfoxaflor 22% AS was diluted approximately 1600 times with water, and each plant was soil-drenched with 6 mL of the solution. Cyantraniliprole at a field rate of 18 g a.i./hm^2^ (converted into a tray rate of approximately 3817 g a.i./hm^2^) was applied as follows: cyantraniliprole 10% OD was diluted approximately 750 times with water, and each plant was soil-drenched with 6 mL of the solution. Water treatment was applied as a water control. All of the above treatments were also tested using two additional methods: in combined with or without white anti-insect nets (50 mesh). The four treatments with net (thiamethoxam & net, sulfoxaflor & net, cyantraniliprole & net, and net alone) were performed in one greenhouse with the net; the other four treatments without net (thiamethoxam-only, sulfoxaflor-only, cyantraniliprole-only, and untreated control) were performed in another greenhouse without the net. The anti-insect net-treated plots were covered with nets from the time of seedling transplanting until the end of the trial. The whole experimental design was briefly descripted in Suppl. Fig. S1.

#### Investigation of B. tabaci eggs and adults

The egg and adult populations of *B. tabaci* on the whole plant were counted on the day of soil drenching, the day of transplanting, and in a random continuous sampling survey after transplanting until the end of the trial, which were also given in Tables 1, 2, 3 and 4, respectively. Ten plants in each plot with four replicates were selected for the egg and adult surveys in the early morning. In order to provide more accurate data, all the leaves of the selected plant were investigated in our two trials. The adult of *B. tabaci* usually lays its eggs on the underside of the leaves, carefully turn the leaves to count egg and adult populations of *B. tabaci* in the early morning with a relatively low temperature to avoid the adults flying away.

#### Determination of insecticide residues in tomato fruits

Tomato fruits in each plot were sampled at the first harvest, and the corresponding insecticide (thiamethoxam, sulfoxaflor and cyantraniliprole) residuals were determined to evaluate the potential safety. The thiamethoxam, sulfoxaflor and cyantraniliprole concentrations were all estimated using liquid chromatography-tandem mass spectrometry (LC–MS/MS). Briefly, 10 g of homogenized tomato fruit sample was added to a 50-mL centrifuge tube, followed by the addition of 10 mL of acetonitrile. The solution was shaken for approximately 10 min, and then 2 g of NaCl and 4 g of MgSO_4_ were added. The solution was then shaken for 3 min and centrifuged for 5 min at 4000 rpm. Finally, 1.5 mL of the resuspended solution was filtered through a micropore membrane filter with a 0.22-µm pore size for analysis using a Waters Xevo TQ-S (Waters Corporation, USA) equipped with an Acquity UPLC BEH C18 column (2.1 m × 100 mm × 1.7 µm). Each sample analysis was carried out in triplicate.

### Statistical analyses

The efficiency of *B. tabaci* adult or egg control was calculated according to the equation^35^:

1 $$\mathrm{Y}=\frac{{\mathrm{X}}_{1}-{\mathrm{X}}_{2}}{{\mathrm{X}}_{1}}\times 100$$

where *Y* is the efficiency of *B. tabaci* adult or egg control, *X*_*1*_ is the number of *B. tabaci* adult or egg per plant in the untreated control, and *X*_*2*_ is the number of *B. tabaci* adult or egg per plant in the treated plots.

Data for *B. tabaci* adult and egg populations were transformed as necessary [square root transformations for small numbers (< 100) and log10 for large numbers (> 100)] for statistical analyses. All of the data are expressed as the means ± SD and were analyzed by ANOVA with SPSS (version 22.0 for Windows, IBM). Significant differences among means were determined by Fisher’s LSD test at *P* = 0.05 level^36,37^.

### Statement

The collection of field studies on plants related to the article "Application of insecticides by soil drenching before seedling transplanting combined with anti-insect nets to control tobacco whitefly in tomato greenhouses" complies with the relevant laws of Mainland China.

## Supplementary Information

Supplementary Information.

## Data Availability

All data generated or analysed during this study are included in this published article.
