# Supplementary material for: Application of insecticides by soil drenching before seedling transplanting combined with anti-insect nets to control tobacco whitefly in tomato greenhouses
Source: Sci Rep. 2022 Sep 24;12:15939. doi: 10.1038/s41598-022-20294-5 (PMC9509379; doi:10.1038/s41598-022-20294-5)
Supplement: Supplementary file 1 — Supplementary Information. [file 41598_2022_20294_MOESM1_ESM.docx]

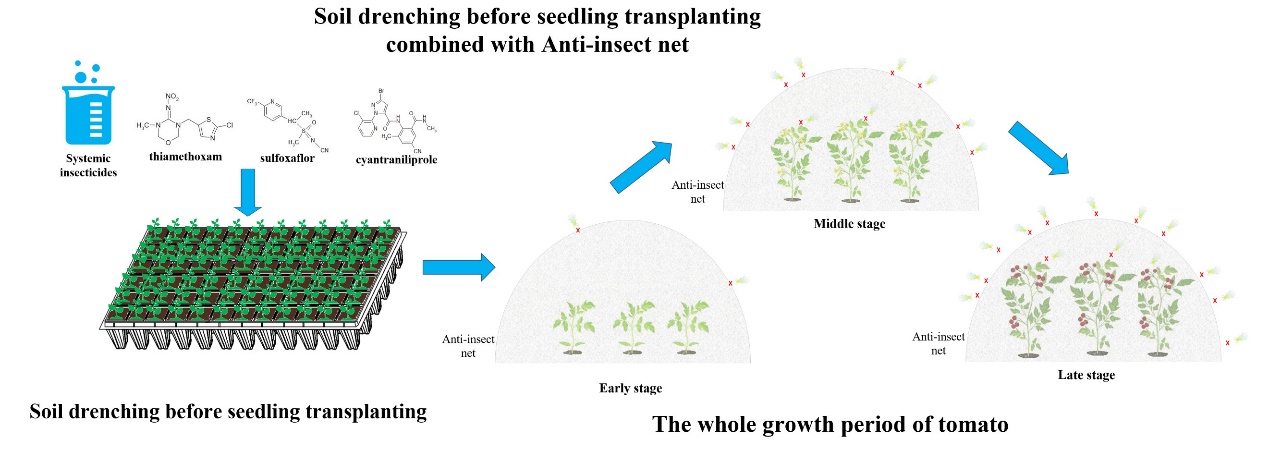


**Figure S1 The insecticides by soil drenching before seedling transplanting combined with anti-insect nets field trials diagram**
